# Whole-Organ CT Perfusion of the Pancreas: Impact of Iterative Reconstruction on Image Quality, Perfusion Parameters and Radiation Dose in 256-Slice CT-Preliminary Findings

**DOI:** 10.1371/journal.pone.0080468

**Published:** 2013-11-26

**Authors:** Qian Xie, Juan Wu, Ying Tang, Yafang Dou, Sijie Hao, Feijia Xu, Xiaoyuan Feng, Zonghui Liang

**Affiliations:** 1 Department of Radiology, Huashan Hospital, Fudan University, Shanghai, China; 2 Department of Radiology, Jing’an District Central Hospital of Shanghai (Jing’an Branch, Huashan Hospital, Fudan University), Shanghai, China; 3 Department of Radiology, Affiliated Hospital of Nantong University, Nantong University, Nantong, China; 4 Department of General Surgery, Huashan Hospital, Fudan University, Shanghai, China; Glasgow University, United Kingdom

## Abstract

**Background:**

This study was performed to assess whether iterative reconstruction can reduce radiation dose while maintaining acceptable image quality, and to investigate whether perfusion parameters vary from conventional filtered back projection (FBP) at the low-tube-voltage (80-kVp) during whole-pancreas perfusion examination using a 256-slice CT.

**Methods:**

76 patients with known or suspected pancreatic mass underwent whole-pancreas perfusion by a 256-slice CT. High- and low-tube-voltage CT images were acquired. 120-kVp image data (protocol A) and 80-kVp image data (protocol B) were reconstructed with conventional FBP, and 80-kVp image data were reconstructed with iDose^4^ (protocol C) iterative reconstruction. The image noise; contrast-to-noise ratio (CNR) relative to muscle for the pancreas, liver, and aorta; and radiation dose of each protocol were assessed quantitatively. Overall image quality was assessed qualitatively. Among 76 patients, 23 were eventually proven to have a normal pancreas. Perfusion parameters of normal pancreas in each protocol including blood volume, blood flow, and permeability-surface area product were measured.

**Results:**

In the quantitative study, protocol C reduced image noise by 36.8% compared to protocol B (P<0.001). Protocol C yielded significantly higher CNR relative to muscle for the aorta, pancreas and liver compared to protocol B (P<0.001), and offered no significant difference compared to protocol A. In the qualitative study, protocols C and A gained similar scores and protocol B gained the lowest score for overall image quality (P<0.001). Mean effective doses were 23.37 mSv for protocol A and 10.81 mSv for protocols B and C. There were no significant differences in the normal pancreas perfusion values among three different protocols.

**Conclusion:**

Low-tube-voltage and iDose^4^ iterative reconstruction can dramatically decrease the radiation dose with acceptable image quality during whole-pancreas CT perfusion and have no significant impact on the perfusion parameters of normal pancreas compared to the conventional FBP reconstruction using a 256-slice CT scanner.

## Introduction

CT perfusion studies can provide anatomical and hemodynamic information of the tissues. Miles et al [1] for the first time applied this technique in pancreas and described the major advantages of combining perfusion information and anatomical details in 1995. Since then, some small sample studies have been conducted to investigate the usefulness of CT perfusion in differentiating the diseased tissue in pancreatic adenocarcinoma, pancreatic endocrine tumors and pancreatitis from normal pancreatic tissue [2], [3]. In most of the previous CT perfusion studies, insufficient coverage was a major limitation due to the detector width, with only part of the organ or larger pancreatic mass covered in the field of view.

Nowadays, with the development of CT scanner, large axial field of detectors can provide up to 16 cm of imaging area for perfusion studies and make the whole-organ perfusion possible. This technique allows the acquisition of perfusion parameters and conventional multiphasic scanning images at the same time.

Although CT perfusion can provide information regarding blood perfusion changes in pancreas, radiation exposure is a persistent problem. In response to the concerns for radiation dose reduction, CT manufacturers have developed some new techniques to help maintain image quality in studies acquired at a lower radiation dose. Recently, a different approach to image reconstruction known as iterative reconstruction (IR) has been used in the clinic. IR techniques use an iterative process to match estimated data to the actual acquired data. The noisiest measurements are given the lowest weight during the IR process and therefore least influence to the final quality of image. Hence, IR techniques treat noise properly at low signal levels, and consequently reduce the noise and artifacts present in the resulting reconstructed image [4], [5]. This results in an overall improvement of image quality and the reduction of radiation dose beyond those achievable with conventional FBP reconstruction. This article describes the first application of this technique to the whole-pancreas CT perfusion studies. Image quality and pancreatic perfusion parameters of each protocol were assessed.

## Materials and Methods

### Ethics

This study was conducted according to the principles expressed in the Declaration of Helsinki and was approved by the Institutional Review Board of Huashan Hospital. Written informed patient consent was obtained from all patients.

### Patients

Seventy-six patients (43 males and 33 females, with ages ranging from 35 to 88 years, mean age 59.1 years) with a clinical suspicion of pancreatic mass based on the results of prior cross-sectional imaging, ultrasonic, magnetic resonance examinations or abnormal tumour marker underwent whole-pancreas CT perfusion at Department of Radiology, Huashan Hospital, Fudan University, Shanghai, China between May 2011 and November 2012 and were included in this study. The patients who were younger than 18 years, in pregnancy or lactation, had any history of anaphylaxis after administration of iodinated contrast agents, known history of renal failure (serum creatinine level > 1.2 mg/dl), or asthma were rejected for use. Of the 76 recruited patients, 31 patients (18 males and 13 females, with ages ranging from 35 to 80 years, mean age 58.6 years) were randomly assigned to the conventional protocol A (120-kVp, 100 mAs), and the remainders (25 males and 20 females, with ages ranging from 36 to 88 years, mean age 59.3 years) were examined with a low radiation protocol B (80-kVp, 100 mAs). The diagnostic information of all 76 patients is shown in Table 1.

**Table 1:** Diagnoses of patients in 120-kVp (protocol A) and 80-kVp (protocol B and protocol C).

Patient diagnoses	120-kVp (n = 31)	80-kVp (n = 45)
Normal pancreas	9	14
Pancreatic adenocarcinoma	13	16
Pancreatic neuroendocrine tumor	4	5
Pancreatic cystadenoma	2	3
Pancreatic pseudocyst	1	1
Duodenal adenocarcinoma	2	4
Duodenal stromal tumor	-	2

Among this population, 23 patients were eventually proven to have a normal pancreas based on negative CT and endoscopic ultrasound findings, normal blood results, and normal follow-up. After diagnosis, these 23 patients (11 males and 12 females, with ages ranging from 36 to 80 years, mean age 55.2 years) were retrospectively included in the normal group. Nine patients (six males and three females, with ages ranging from 38 to 80 years, mean age 56.0 years) received protocol A CT scans and 14 patients (five males and nine females, with ages ranging from 36 to 78 years, mean age 54.7 years) received protocol B CT scans. The mean body mass index [BMI  =  weight (kg) divided by the square of height (m)] of all included 76 patients was 23.1±2.0 kg/m^2^(ranging from 17.5 to 27.4 kg/m^2^).The BMI of patients who received protocols A or B CT scans was 23.5±1.8 kg/m^2^ and 22.8±2.2 kg/m^2^, respectively.

### Perfusion CT Technique

Whole-pancreas CT perfusion was performed with a 256-silce CT scanner (Brilliance iCT; Philips Medical Systems, Cleveland, OH, USA). Before examination, all patients received 800 mL water perorally to distend the stomach and duodenum for a better delineation of the pancreas. Patients were given abdominal belt to reduce the artifacts caused by respiratory motion. Before examination, they were trained to breathe shallowly and regularly to minimize respiratory excursions of the abdominal wall. All patients were asked to lie in the supine position, with the feet on the scanning table. Then, a craniocaudal localization topogram was acquired (100 kVp, 30 mAs), and an unenhanced scan (120 kVp, 100 mAs, 5 mm slice thickness, 0.9 mm pitch, 0.5 s rotation time) was performed to make sure that the whole pancreas would be covered by the 16-cm perfusion imaging field. Using a high pressure syringe, 50 mL of nonionic contrast agent (Omnipaque, 350 mg iodine/mL; GE Healthcare, Shanghai, China) was intravenously administered at a flow rate of 5 mL/s through a 21-gauge catheter, followed by a saline flush of 30 mL (5 mL/s) serving as a bolus chaser. CT perfusion examinations were then performed in a volume scanning pattern, 80 kVp (protocol B) or 120 kVp (protocol A), 100 mAs, 5 mm slice thickness, field-of-view 350 mm, 512×512 pixels.

The protocol for whole-pancreas CT perfusion contained of a total of 13 intermittent volume acquisitions, which were acquired over a time period of 85 seconds. A volume acquisition was performed every 7 seconds: 2 seconds of scanning time and 5 seconds of interval time. After completion of image acquisition of the 13 series, images of the fourth series were chosen for quantitative image quality analysis, because the acquisition time (28 seconds after contrast medium injection) corresponded approximately to the optimal pancreatic parenchymal phase images and offered the greatest likelihood of pancreatic tumor depiction[6], [7].

### CT Image Reconstruction

The120-kVp (protocol A) and 80-kVp (protocol B) CT image data were reconstructed with conventional FBP algorithm. The same 80 kVp raw data were reconstructed into protocol C image sets using the iDose^4^ algorithm (Philips Healthcare) (Table 2).

**Table 2:** CT scanning parameters and postprocessing algorithms of protocol A, B, and C.

Parameter	Protocol A	Protocol B	Protocol C
Detector configuration (no. of sections×mm)	128×0.625	128×0.625	128×0.625
Voltage (kVp)	120	80	80
Tube current-time product (mAs)	100	100	100
Slice thickness (mm)	5	5	5
Rotation time (sec)	0.5	0.5	0.5
Scanning time (no. of acquisitions×sec)	13×7	13×7	13×7
Reconstruction algorithm	FBP	FBP	iDose^4^

IDose^4^ is an IR technique. This IR algorithm allows the user to adjust the image noise level using a parameter called “iDose^4^ level”: the higher the iDose^4^ level, the greater the noise reduction. The machine offers seven levels to control or reduce the amount of image noise at a given tube output [8], [9]. On the basis of manufacturer suggestion and our preliminary clinical experience, we chose level 4(iDose^4^level 4) to image reconstruction.

### Qualitative Image Quality Analysis

Two experienced radiologists (Z.-H.L. and T.Y. with 18 and 6 years of experience in abdominal radiology, respectively) blinded to the specific image reconstruction technique, assessed all images independently. The image sets acquired with the three protocols were intermixed and presented to the two radiologists in a randomized order. Neither of the radiologists was involved in the scanning process. We analyzed this qualitative image at a window width and level of 40 and 280 HU, respectively (standard abdominal window settings). The evaluation criteria used a 5 point Likert scale: a score of 5: Image quality considered superior, with obvious lack of image noise and absence of any artifact or distortion; 4: image quality considered very good, showing image noise typically encountered in our daily clinical practice, and not compromised by artifact or distortion of spatial resolution or contrast resolution; 3: image quality considered good and only minimally compromised by image noise, artifact, or minimal distortion of contrast or spatial resolution; 2: image quality considered fair but significantly compromised by moderate image noise, some image artifact, or some distortion of contrast or spatial resolution; 1: image quality considered poor and non-evaluable because of high image noise, marked artifact, distortion of contrast resolution or spatial resolution, or poor edge definition. Radiologists were instructed to use score of 3, 4, or 5 if they considered an image set diagnostically acceptable in a clinical setting and to use score of 1 or 2 if diagnostically unacceptable[10], [11]. Images were viewed at GE PACS workstations. To prevent the reviewers from recognizing the test pattern within the image, all images were de-identified of clinical information and reconstruction type and were only shown once.

### Quantitative Image Quality Analysis

A radiologist (X.Q, with 6 years of experience in abdominal radiology) performed quantitative measurements of 5-mm thick transverse images. The three image sets, obtained with protocols A, B, and C were displayed with standard abdominal window settings (window level, 40 HU; window width, 280 HU).

**Figure 1:**
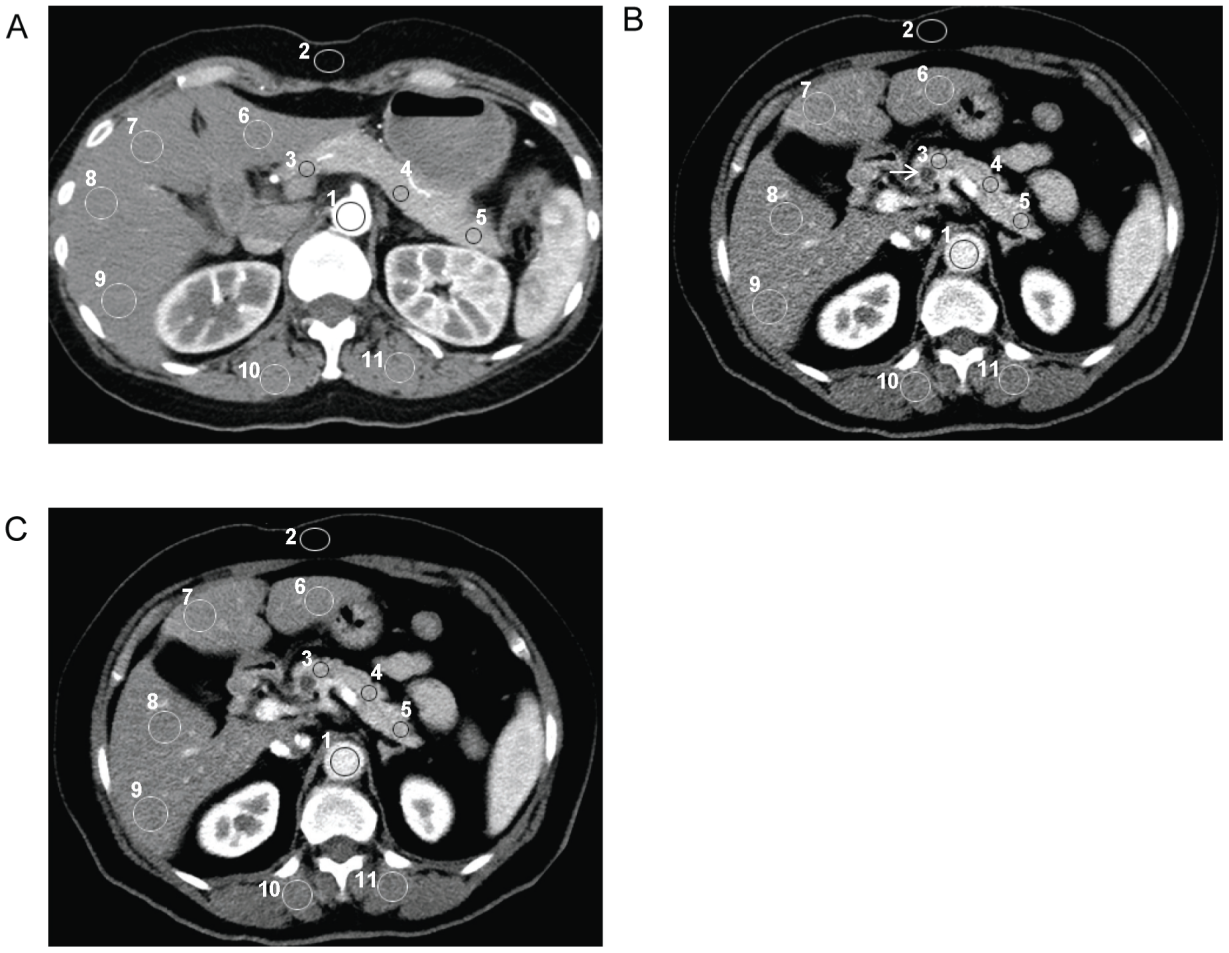
Conventional transverse pancreas CT perfusion images. (A) Protocol A (120-kVp, filtered back projection) in a 48-year-old female patient. Images from a 66-year-old female patient showing a cystic lesion in the head of the pancreas (arrow), with (B) protocol B (80-kVp, filtered back projection), and (C) protocol C (80-kVp, iDose^4^), which show region of interests (ROIs) manually drawn on aorta (ROI 1), subcutaneous fat of anterior abdominal wall (ROI 2), pancreas (ROI 3-5), liver (ROI 6-9), paraspinal muscle (10,11). For all measurements of the same patients, size, shape, and position of ROIs were kept constant by applying copy-and-paste function at workstations.

The mean CT attenuation values (in Hounsfield units) of the aorta, pancreas, liver, and bilateral paraspinal muscles were measured by manually placing circular regions of interest (ROIs) (Fig 1). The attenuation of the aorta was recorded from a single drawn ROI (mean number of pixels, 250; range, 150–400 pixels) that was not so small as to be affected by pixel variability and not so large to include the calcifications and soft plaques of the aortic wall. The attenuation of the pancreas was recorded as the mean measurement of three ROIs (mean number of pixels, 100; range, 50–250 pixels) on the pancreatic head, body, and tail. Areas of focal changes in large vessels, pancreatic ducts, and prominent artifacts were carefully avoided. The attenuation of the hepatic parenchyma was recorded as the mean measurement of four ROIs (mean number of pixels, 400; range, 250–600 pixels) placed in the right anterior, right posterior, left medial and left lateral segments of the liver. Visible blood vessels, bile ducts, parenchymal attenuation, and artifacts were carefully avoided. The attenuation of the bilateral erector spinae muscle was measured from two ROIs (mean number of pixels, 400; range, 300–800 pixels) while excluding macroscopic areas of fat infiltration. In addition, the image noise of each protocol was measured as the standard deviation of the pixel values from a circular ROI (mean number of pixels, 250; range, 100–400 pixels) drawn in a homogeneous region of the subcutaneous fat of the anterior abdominal wall. The size, shape, and position of the ROIs were maintained constant among the protocols B and C by applying a copy and paste function at the workstation. To ensure consistency, all measurements were conducted three times at the level of the main portal vein on three adjacent images, and mean values were computed. For each of three protocols, CNR relative to muscle for the aorta, pancreas, and liver was calculated by the equation: CNR = (ROI_organ_– ROI_muscle_)/SD_noise_, where the ROI_organ_ is the mean attenuation of the organ at interest, and the ROI_muscle_ is the mean attenuation of the erector spinae muscles, and SD_noise_ is the mean image noise[12].

### Perfusion Data Processing

Among all 76 patients who accepted whole-pancreas CT perfusion examination, 23 were eventually proven to have a normal pancreas. For the normal group in three protocols (protocol A_normol_: 9 patients; protocol B_normal_ and protocol C_normal_: 14 patients), image data were processed using a workstation with professional perfusion software (MIStar, Version 3.2; Apollo Medical Imaging Technology Pty. Ltd; North Melbourne, VIC, Australia) by a radiologist (X.Q.) with 6 years of experience in abdominal radiology. The parametric maps and perfusion parameters including blood flow (BF, mL/100 g/min), blood volume (BV, mL/100 g), and permeability surface area product (PS, mL/100 g/min) were calculated and analyzed using deconvolution method.

**Figure 2:**
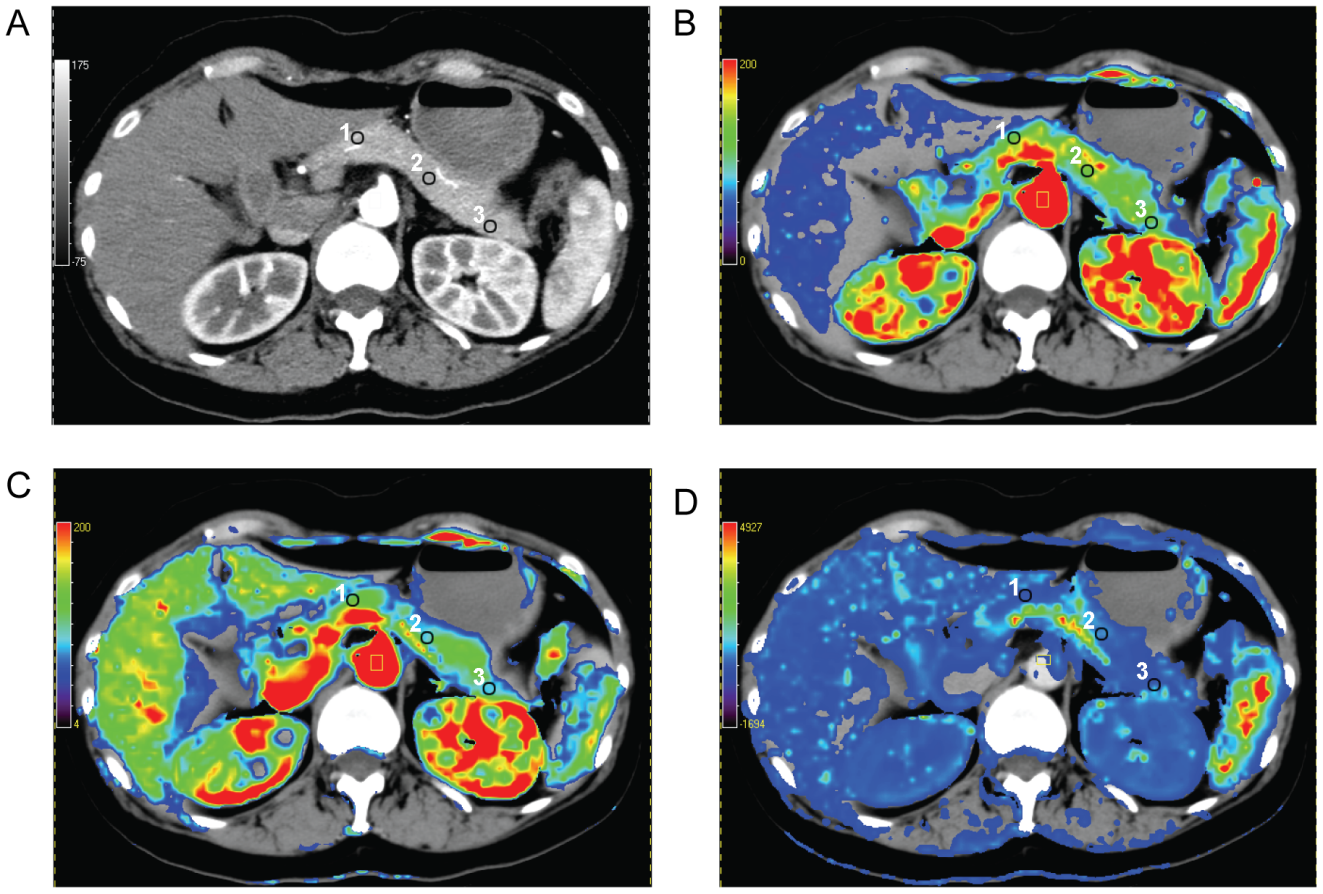
Functional perfusion CT color maps of normal pancreatic tissue in a 48-year-old female. (A) Transverse CT image. (B-D) CT perfusion parametric maps for blood flow, blood volume, and permeability-surface area product. Regions of interest (ROIs) drawn on pancreatic head (ROI 1), body (ROI 2), and tail (ROI 3). For all measurements of the same patient, size, shape, and position of ROIs were kept constant by applying copy-and-paste function at workstations.

The arterial input was set by manually placing a circular ROI on the abdominal aorta. An arterial time-enhancement curve for the 85-second acquisition time was generated automatically as well as parametric maps of BF, BV, PS. A total of three ROIs (mean number of pixels, 50; range, 30–100 pixels) of each case were then drawn in the head, body, and tail of normal pancreatic tissue of the parametric perfusion maps (Fig 2), and mean values were computed. Care was taken to avoid adjacent normal vasculature and pancreatic duct. A tissue time-enhancement curve and the three perfusion parameters (BF, BV, and PS) within the ROIs were derived or selected. Three perfusion parameters of ROI were recorded for each patient with normal pancreas.

### CT Radiation Dose

To assess effective whole-pancreas perfusion CT radiation doses, we recorded the CT dose index (CTDI_vol_) and dose-length product (DLP). The effective radiation doses for the three acquisitions were derived from the product of the DLP and the conversion coefficient for the abdomen (k = 0.015 mSv×mGy^−1^×cm^−1^) [11].

### Statistical Analysis

Data were analyzed using statistical software (SAS, version 9.2; SAS Institute Inc, Cary, NC, USA). Results were expressed as mean ±standard deviations. The differences in image noise and CNR among the three protocols were assessed, and one-way analysis of variance was conducted, with protocols serving as the analysis of variance factors.

The estimates from this model were compared in paired fashion using the Bonferroni adjustment for the multiple comparisons. For the group of normal pancreas in three protocols, one-way analysis of variance followed by Bonferroni method of multiple comparisons was used to calculate variations in CT perfusion parameters. For qualitative evaluation offered by two radiologists, nonparametric Friedman test was applied to assess statistically significant differences in image quality scores and Steel-Dwass test was performed for pairwise comparisons. A P value of less than 0.05 was considered statistically significant. Interobserver agreement was evaluated by using the Kappa test [13]. The scale for K coefficients for interobserver agreement was as follows: less than 0.20, poor agreement; 0.21–0.40, moderate agreement; 0.41–0.60, fair agreement; 0.61–0.80, substantial agreement; and 0.81– 1.00, almost perfect agreement.

## Results

### Patients

There were no significant differences between the 80-kVp and 120-kVp groups with respect to age and body mass index.The normal population in two groups also showed no significant difference with respect to age and body mass index.A summary of patient characteristics is shown in Tables 3 and 4.

**Table 3:** Characteristics of patients in 120-kVp (protocol A) and 80-kVp (protocol B and protocol C).

Patient characteristics	120-kVp	80-kVp	P value
No. of patients	31	45	
Age (yr)	58.6±12.2	59.3±12.4	0.8111
Body mass index (kg/cm^2^)	23.5±1.8	22.8±2.2	0.1302

Unless otherwise specified, data are expressed as mean±standard deviation.

**Table 4:** Characteristics of patients with normal pancreas in 120-kVp (protocol A) and 80-kVp (protocol B and protocol C).

Patient characteristics	120-kVp	80-kVp	P value
No. of patients	9	14	
Age (yr)	56.0±14.9	54.7±12.5	0.9256
Body mass index (kg/cm^2^)	23.8±1.6	23.3±2.2	0.5755

Unless otherwise specified, data are expressed as mean±standard deviation.

### Qualitative Image Quality Analysis

For all 76 patients who accepted whole-pancreas CT perfusion examination, the mean scores of image quality evaluated by the two radiologists are shown in Table 5. Both radiologists graded a similar score between protocol A and protocol C (3.8 and 3.6). There was no significant difference between protocol A and protocol C, with the overall image quality for both protocols in the diagnostically acceptable range. Meanwhile, the lowest score was marked for protocol B (2.6), with the overall image quality in the diagnostically unacceptable range. The mean score of protocol B was significantly lower than the other two protocols. Interobserver agreements on visual scores were almost perfect (Cohen’s kappa = 0.85).

**Table 5:** Subjective image quality scores for protocols A, B, and C from the two radiologists (reader 1 and reader 2).

		Reader 1			Reader 2		Cohen’s
	Protocol A	Protocol B	Protocol C	Protocol A	Protocol B	Protocol C	Kappa**
Image quality score	3.80	2.66	3.64	3.80	2.62	3.62	0.8539
P value	<0.05*	<0.05*	>0.05	<0.05*	<0.05*	>0.05	
	A vs B	B vs C	C vs A	A vs B	B vs C	C vs A	

Data are expressed as mean scores. *Value shows statistical difference. **Value was reported as follows: poor, less than 0.20; fair, 0.21–0.40; moderate, 0.41–0.60; substantial, 0.61–0.80; and almost perfect, 0.81–1.00.

### Quantitative Image Quality Analysis

Mean image noise was significantly lower with protocol A (9.5 HU) and protocol C (10.6 HU) than with protocol B (16.9 HU) (P<0.001, for both comparisons). The mean CNRs of aorta, pancreas, and liver were significantly higher with protocol C (29.2±9.8, 5.4±1.8, 1.9±0.8, respectively) than with protocol B (18.3±5.6, 3.6±1.5, 1.2±0.5, respectively).Meanwhile, there was no significant difference in mean CNR values between protocol C and protocol A (31.2±6.5, 5.4±1.2, 1.8±0.7, respectively), and both of two protocols yielded significantly higher mean CNR values when compared with protocol B (P<0.001, for both comparisons) (Table 6).

**Table 6:** Contrast-to-noise ratio (CNR), image noise and radiation dose for protocols A, B, and C.

					P Value	
CT parameter	Protocol A	Protocol B	Protocol C	Protocol C vs.	Protocol C vs.	Protocol B vs.
				protocol A	protocol B	protocol A
CNR						
Aorta	31.18±6.51	18.25±5.55	29.18±9.77	0.2629	<.0001	<.0001
Pancreas	5.35±1.23	3.56±1.48	5.43±1.80	0.8218	<.0001	<.0001
Liver	1.83±0.73	1.18±0.46	1.88±0.76	0.7238	<.0001	<.0001
Image noise (HU)	9.54±0.67	16.86±1.27	10.66±0.93	<.0001	<.0001	<.0001
CTDIvol (mGy)	4.51±0.41	1.97±0.16	1.97±0.16	<.0001	-	<.0001
DLP (mGy.cm)	1557.71±138.78	720.58±47.29	720.58±47.29	<.0001	-	<.0001
Effective dose (mSv)	23.37±2.1	10.81±0.71	10.81±0.71	<.0001	-	<.0001

Data are expressed as mean±standard deviation.

### Perfusion Parameters

**Figure 3:**
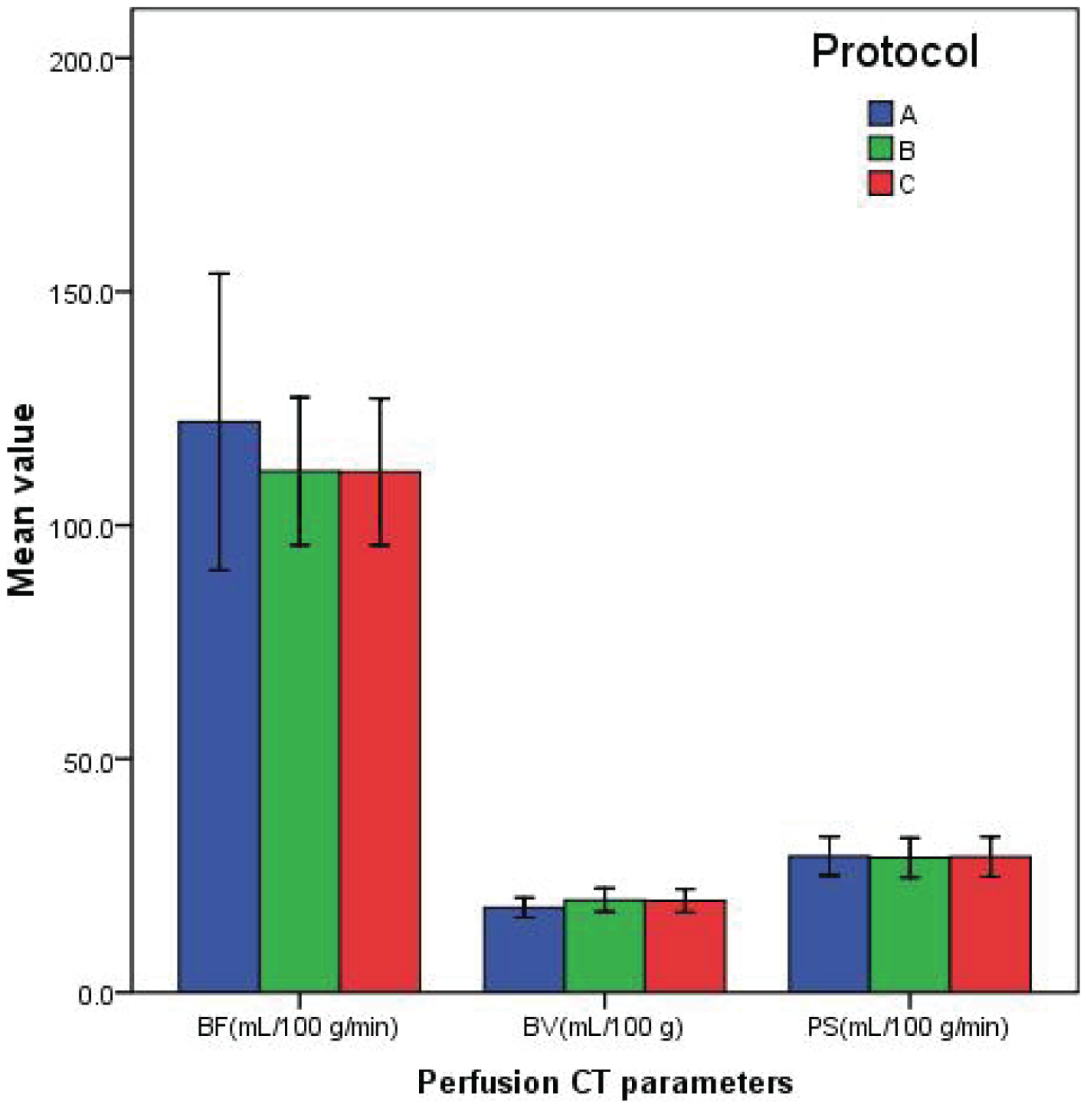
Bar graph of perfusion CT parameters of normal pancreas in the three protocols. Blood flow (BF), blood volume (BV), and permeability-surface area product (PS). Means (standards deviations) are indicated in the figure.

There were no significant differences in the normal pancreas perfusion values (BF, BV, and PS) among three different protocols (P>0.05) (Fig 3). The results for BF, BV, and PS of different protocols in confirmed normal pancreas are shown in Table 7.

**Table 7:** Measurements of blood flow (BF), blood volume (BV), and permeability-surface area product (PS) in three protocols with normal pancreas.

Perfusion parameters	Protocol A	Protocol B	Protocol C	P value
BF (mL/100 g/min)	122.13±31.77	111.55±15.82	111.42±15.70	0.4145
BV (mL/100 g)	18.17±2.12	19.79±2.54	19.64±2.48	0.2606
PS (mL/100 g/min)	29.19±4.12	28.85±4.21	29.05±4.27	0.9913

Data are expressed as mean±standard deviation.

### Radiation Dose

During the whole-pancreas perfusion CT, the mean CTDI_vol_ for protocols B and C (1.97±0.16 mGy) was significantly less than for the protocol A (4.51±0.41 mGy, 56.3% less) (P<0.001). The mean DLP for protocols B and C (720.58±47.29 mGy.cm) was significantly lower than that for protocol A (1557.71±138.78 mGy.cm, 53.8% less) (P<0.001). Likewise, the mean effective radiation dose for protocols B and C (10.8±0.7 mSv) was significantly lower than that for protocol A (23.4±2.1 mSv, 53.8% less) (P<0.001) (Table 6), comparable to and even lower than the total dose during a upper abdominal triphasic enhanced CT scan[5], [11], [14], [15].

## Discussion

CT perfusion is a kind of functional imaging that reflects the hemodynamic changes of tissues in addition to the morphological changes by measuring perfusion parameters of tissues and lesions [16], [17]. Merier and Zierler had raised its principles back in 1954, but it was not put into clinical research due to the simplicity of CT equipment at that time. Until 1991, the fundamental theory of CT perfusion was raised by Miles on the basis of indicator dilution theory and central volume principle. It was acknowledged as the landmark moment for the development of CT perfusion [18], [19]. Since then, this technique has been gradually applied to the brain, heart, and liver. Especially in the brain, CT perfusion plays a crucial role in the early detection of acute stroke and directly impacts the therapeutic interventions [20]–[22]. Nowadays, brain CT perfusion has been widely applied in the clinic such as use in assessment of the degree of ischemia and differentiation between cerebral infarction and cerebral ischemia [23]–[25]. In addition, by providing an in vivo marker of tumor angiogenesis, CT perfusion can be used for diagnosis and therapeutic monitoring of intracranial neoplasms [26], [27].

Miles for the first time applied CT perfusion technique to the pancreas in 1995 [1], and soon afterwards, some small sample studies have been conducted in droves. However, bottlenecks were encountered in the research of pancreatic CT perfusion throughout the world [14]. Two major problems blocked the development of pancreatic CT perfusion and even hindered the advancement in the entire perfusion research. First, current multi-slice CT equipment for clinical application usually had limited coverage of detectors (64-slices CT: 4 cm width of detectors), neither the whole pancreas from the range of head to tail nor the large size neoplasms could be imaged completely during CT perfusion [28].

But now, a recently introduced 256-slice CT equipment covers an anatomical region of 16 cm in the isocenter of gantry, so whole-organ perfusion of the pancreas comes into reality. CT perfusion has advanced significantly, i.e., from lesion perfusion to organ perfusion. Secondly, radiation exposure-accompanied CT perfusion scanning is a continuous concern due to its potential harm to patients. The newly developed iterative reconstruction technique allows dose reduction while maintaining acceptable image quality by performing repeated iterative reconstruction cycles. Previous studies using iterative reconstruction techniques in chest CT, abdominal CT and coronary CT angiography have shown promise for radiation dose reduction while maintaining or even enhancing image quality [29]–[33].

Our study for the first time combined the iterative reconstruction with the whole-organ perfusion technique of the pancreas using advanced 256-silce CT. Our protocols aimed at acquiring the whole-pancreas perfusion parameters and routine enhanced CT images during a single perfusion scan. In order to maintain the image quality obtained by our low kV protocol (80 kVp), we tentatively applied the iDose^4^ algorithm to whole-pancreas perfusion scanning to evaluate whether the overall image quality corresponded approximately to the standard dose setting perfusion scanning (120 kVp) in a previous study [28].

In this study, we found that image reconstruction of low dosage (80 kVp) CT data of whole-pancreas perfusion with iterative reconstruction algorithm (iDose^4^ level 4) yielded significant improvements in image noise, CNR and image quality compared with FBP algorithm. The use of 256-slice CT with 80 kVp and iDose^4^ algorithm yields little difference in image noise, CNR and visual score during whole-pancreas perfusion CT despite a 53% reduction in the radiation exposure (protocol C, 10.8±0.7 mSv; protocol A, 23.4±2.1 mSv, respectively), compared with the conventional 120 kVp protocol. The overall radiation dose (including the localization topogram and unenhanced scan) deployed by our low kV CT perfusion corresponded approximately or even lower than the dose of a triphasic upper abdominal imaging procedure [5,11 14,15]. Nevertheless, whole- pancreas perfusion parameters and conventional axial images that could be used in clinical diagnosis were acquired during a single scan. Our visual evaluation showed a corresponding result that the overall image quality of 80 kVp iDose^4^ protocol was significantly improved compared with the 80 kVp FBP protocol, furthermore, showed slightly, but not significantly lower diagnostically acceptable image quality compared with the 120 kVp FBP protocol.

With respect to the whole-pancreas CT perfusion, we assessed three main perfusion parameters (BF, BV, and PS). BF represents the volume of blood that flows in a fixed quantity of tissue per unit time. BV reflects the amount of BF in the local regional tissue, and is influenced by the size of the afferent vessels and number of capillaries that open up. PS indicates the rate of unidirectional flow of contrast agent via the blood capillary endothelium to interstitial spaces [1], [21]. The use of 80 kVp iDose^4^ protocol offered almost the same BF, BV, and PS values compared to the 80 kVp FBP protocol. There were also no significant differences in BF, BV, and PS values between the 80 kVp protocol and 120 kVp protocol. That was because perfusion parameters were calculated not primarily based on the diagnostic image quality of every individual perfusion series, but rather on the density change over a ROI.

There were several potential limitations in our study. First, because this study was a part of assessment of tumor angiogenesis in pancreatic adenocarcinoma using 256-silce perfusion CT, the majority of patients enrolled in our study had relatively small to medium body habitus with a BMI less than 25 kg/m^2^. In patients with large size body, 80 kVp perfusion CT tends to yield conventional axis images of poorer quality that cannot be used in clinical diagnosis because of the increased image noise [34], [35]. Thus, the findings of our study should be applied with caution to the obese patients. Second, although no significant differences in perfusion parameters were observed between three protocols (120 kVp FBP, 80 kVp FBP, 80 kVp iDose^4^), the current study findings reflect our preliminary experience with a small number of patients. Therefore, further studies need to be performed to validate our findings. Third, another limitation was that healthy pancreas perfusion parameters of 80 kVp and 120 kVp protocols were not obtained from the same patients. Although both groups with healthy pancreas had similarly average age and BMI, the problem may still limit the generalization of our findings. It is immoral to scan a patient twice just for testing so further animal experiments should be conducted to confirm our encouraging findings.

## Conclusions

In conclusion, our preliminary study shows that compared to the conventional FBP reconstruction technique, iDose^4^ iterative reconstruction technique yields a significant improvement in image quality and appears not to impede calculation of healthy pancreatic perfusion parameters, but helps to constrain the radiation dose of whole-pancreas CT perfusion within a commonly accepted level in upper-abdominal imaging.
